# The Limited Significance of the Internal Rotation Stress Test in Pediatric Gartland Type III Supracondylar Humerus Fractures

**DOI:** 10.3390/jcm14072276

**Published:** 2025-03-26

**Authors:** Sungmin Kim, Jun-Hyuk Lim, Myung-Jin Sung, Hyeon-Su Na, Gyo-Rim Kang, Sung-Taek Jung

**Affiliations:** 1 Department of Orthopedic Surgery, Chonnam National University Medical School and Hospital, 42 Jebong-ro, Dong-gu, Gwangju 61469, Republic of Korea; smkimos@jnu.ac.kr (S.K.); ove03@naver.com (J.-H.L.); smj2383@naver.com (M.-J.S.); blesha16@gmail.com (H.-S.N.); 2 Department of Orthopedic Surgery, Chonnam National University Hospital, 42 Jebong-ro, Dong-gu, Gwangju 61469, Republic of Korea; gyorim0120@gmail.com

**Keywords:** supracondylar fracture, pediatric, internal rotation stress test, Gartland

## Abstract

**Background:** Gartland type III pediatric supracondylar humerus fractures can be unstable and prone to loss of reduction. The Internal Rotational Stress Test (IRST) aims to assess and address rotational instability during surgery. **Method:** This retrospective study analyzed treatments for Gartland type III pediatric supracondylar humerus fractures at our institution from January 2020 to December 2022. Only patients who underwent IRST were included. IRST was performed after inserting either two or three lateral pins. Patients were divided into Group 1 (IRST +) or 2 (IRST −) based on IRST results. Radiographic and clinical outcomes were compared between the two groups. **Result:** A total of 46 patients were included in the study. The mean age at the time of diagnosis was 5.7 years (range, 4 to 11 years), and the mean duration of follow-up was 2.8 years (range, 1.0 to 4.8 years). Group 1 consisted of 24 patients, and Group 2 comprised 22 patients. We did not find any differences in radiographic parameters and clinical scores between the two groups. Additionally, in both groups, there were no instances of major loss of reduction, defined as greater than 12 degrees or 12%. In five patients, we identified two types of fracture patterns that were stable with only two lateral pins. **Conclusions:** In patients with Gartland type III supracondylar humerus fractures, if reduction is adequately achieved and sufficient fixation force is maintained, the IRST results do not significantly impact radiologic and clinical outcomes. The pattern of the fracture can influence instability, necessitating further research on this matter.

## 1. Introduction

Pediatric supracondylar humerus fractures are among the most common elbow injuries in children and are a predominant orthopedic concern in children, known for their complexity and potential for significant complications [1,2,3]. Loss of reduction at the medial column is an impactful complication that can lead to the need for revision fixation and/or malunion with cubitus varus deformity [2,4,5].

The internal rotational stress test (IRST), was initially proposed by Zenios et al. [6], is a procedure designed to identify and address rotational instability during surgery, potentially preventing postoperative complications such as loss of reduction or malunion. Zenios et al. [6] described the use of the IRST in their paper as follows: Initially, two lateral pins are inserted. Subsequently, an IRST is conducted. If lateral instability is observed under the lateral C-arm, a third lateral pin is added. Afterward, the IRST is repeated. If instability persists, additional medial pins are inserted. This test influences the decision to insert medial pins alongside the standard lateral pins to enhance fracture stabilization.

The utility of IRST in guiding the decision for additional pinning has been supported by recent studies [5,7,8].

However, the guidelines provided by the IRST regarding whether an additional pin or a medial pin should be inserted are not clear. Additionally, these guidelines are not consistent across studies dealing with the IRST [6,9]. Moreover, the IRST is a kind of stress test; even if a supracondylar fracture is well reduced and securely fixed, internally rotating the elbow during the test could potentially disrupt the fixation, and the force applied may not be consistent.

The status of reduction and proper power of fixation are crucial for a favorable outcome. We determine appropriate fixation power by assessing C-arm anteroposterior (AP) and external rotational lateral views. If these views are acceptable, we judge the fixation to be adequate and do not insert an additional pin. Even if the IRST is positive, we assume it would not significantly impact postoperative outcomes if the two views are acceptable.

We aimed to investigate whether there are differences in clinical and radiological outcomes based on IRST results.

## 2. Materials and Methods

The study, approved by The Institutional Review Board of the Chonnam National University Hospital (IRB No. CNUH-2024-091), retrospectively analyzed medical records of patients treated for pediatric supracondylar humerus fractures at our institution from January 2020 through December 2022. The inclusion criteria were (1) diagnosis of Gartland classification type III supracondylar humerus fractures [10], (2) a minimum of one year of follow-up post-surgery, (3) patients who demonstrated appropriate reduction and fixation in C-arm AP and external lateral views, and (4) patients who underwent the IRST during surgery. Patients were excluded if an open reduction was required or if inadequate radiographs were available to measure all the radiographic parameters. Data, including patient age at the surgery, sex, follow-up duration, and complications related to the surgical procedure, were collected.

**Figure 1:**
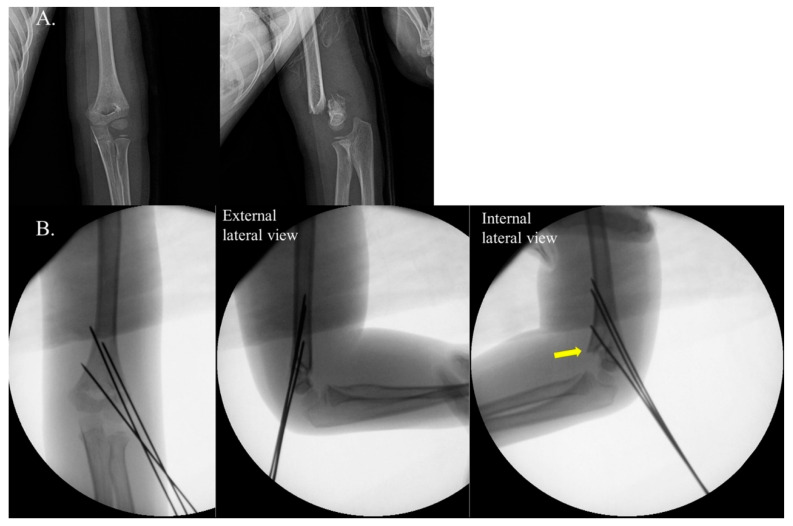
Example of positive internal rotational stress test (IRST) results in a 5-year-old boy with a Gartland type III supracondylar fracture. Panel (**A**) shows the preoperative radiographs. Panel (**B**) depicts C-arm images demonstrating a positive IRST. Good bone contact and well-maintained reduction are observed in both the anteroposterior (AP) and external lateral views. However, note (yellow arrow) the loss of reduction due to fragment rotation in the internal stress lateral view.

**Figure 2:**
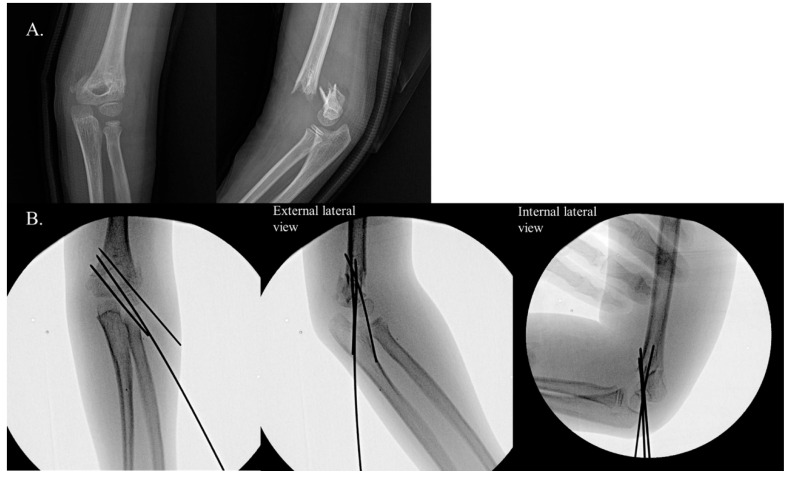
Example of negative internal rotational stress test (IRST) results in a 9-year-old boy with a Gartland type III supracondylar fracture. Panel (**A**) shows the preoperative radiographs. Panel (**B**) depicts C-arm images demonstrating a negative IRST. Good bone contact and well-maintained reduction are observed in both the anteroposterior (AP) and external lateral views. Notably, the reduction was maintained even in the internal stress lateral view.

Based on the intraoperative assessment of rotational instability using the IRST, patients were categorized into two groups: Group 1 (Figure 1): This group included cases where rotational instability was identified by IRST during surgery; Group 2: This group consisted of cases without any evidence of rotational instability intraoperatively (Figure 2).

Initially, two lateral pins were inserted. If the AP view or external rotation lateral view on the C-arm was deemed unacceptable, an additional lateral third pin or a medial pin was inserted. If there is any concern about the fracture stability or whether the pin placement is optimal, a third pin is placed from the lateral side as well. We tried to insert a lateral third pin whenever possible in cases with Gartland type 3, as stability can be a concern [3,11]. In instances where the fracture configuration did not permit the insertion of a third lateral pin, a medial pin was inserted. A 1 cm incision was always made when inserting the medial pin to ensure that the ulnar nerve was not involved under direct visualization [12]. We then assessed the reduction status by confirming acceptable alignment using C-arm AP and external lateral views. If both views were acceptable, no additional pins were inserted. IRST was performed after the insertion of either two or three lateral pins. Even if the IRST yielded a positive result, indicating potential instability, we did not proceed with additional pin fixation if the two views were acceptable.

**Figure 3:**
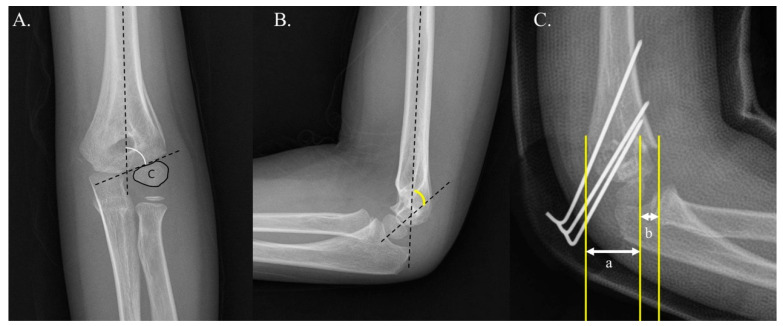
Panel (**A**) demonstrates how the Baumann angle can be measured on the anteroposterior view of radiographs. The Baumann angle is formed by the axis of the humerus and a line drawn through the epiphyseal plate of the capitellum. Panel (**B**) shows how the humerocapitellar angle, consisting of longitudinal lines along the diaphysis of the humerus and the axial line of the capitellum, can be measured on the lateral image. Panel (**C**) illustrates the method for measuring the lateral rotational percentage. The displacement (a) is measured and then divided by the width of the humerus distal to the fracture site (b). This ratio is subsequently multiplied by 100 to yield the percentage of rotation.

The data were analyzed to discern any differences in surgical outcomes between Group 1 and Group 2. Both radiographic and clinical parameters were used for assessment. The radiographic evaluation was based on the Baumann angle [13], humerocapitellar (HC) angle [14,15], and lateral rotational percentage [9] (Figure 3). These angles in coronal, sagittal, and rotational alignment were considered to assess the quality of fracture reduction and to determine the occurrence of loss of reduction. A comparative analysis was conducted between immediate post-surgery values and those obtained at the three-month follow-up. A loss of reduction was defined as a deviation greater than 12 degrees in the Baumann or humerocapitellar angles, or a change greater than 12% in the lateral rotational percentage [11,16,17,18,19].

Clinical Assessment: Functional outcomes were measured using Flynn’s score [20], which is recognized for its effectiveness in evaluating the functional outcomes of pediatric elbow injuries. This score encompasses parameters such as pain, range of motion, and carrying angle. Clinical outcomes were assessed by comparing the immediate post-surgical scores with those obtained one year post-surgery, providing insights into the long-term functional recovery of the patients.

### Statistical Analysis

All continuous variables were expressed as the mean with a range. All continuous variables were tested for their normal distribution by the Shapiro–Wilk test and were confirmed as such. Continuous variables, such as the radiographic measurements, were analyzed using independent *t*-tests or Mann–Whitney U tests, depending on the data distribution. Categorical variables, including Flynn’s score, were compared using Chi-square tests or Fisher’s exact tests, as appropriate for the sample sizes. A *p*-value of less than 0.05 was considered statistically significant. Statistical analysis was performed using SPSS software (version 26.0, SPSS; Chicago, IL, USA).

## 3. Results

Out of the 144 patients initially assessed, 46 met the inclusion criteria and were ultimately included in the study. Exclusions included two cases with insufficient follow-up, sixty-three with Gartland type I or II fractures, six cases with open reduction, and twenty-seven with unclear documentation regarding the performance of the IRST in the medical records.

Of the 46 patients included in the study, 26 (56.5%) were male. The mean age at the time of diagnosis was 5.7 years (range, 4 to 11 years), and the mean duration of follow-up was 2.8 years (range, 1.0 to 4.8 years). In terms of the affected limb, the left side was involved in 18 cases, while the right side was impacted in 28 cases.

Group 1 consisted of 24 patients, and Group 2 comprised 22 patients. The mean age for Group 1 was 5.95 years (range, 4–8) and, for Group 2, it was 6.21 years (range, 4–19); there was no statistically significant difference between the two groups (*p* = 0.496). The pin configurations for each group of patients are described in Table 1. There were five patients in Group 1 who received two lateral pins, with no instances in Group 2. Medial pin fixation was performed on ten patients, all of whom were in Group 2.

We did not find any differences in the radiographic parameters and clinical scores between the two groups (Table 2 and Table 3). In addition, in both groups, there were no instances of major reduction loss, defined as greater than 12 degrees or 12%.

We conducted a subgroup analysis to evaluate the stability of fixation based on the pin configuration (Table 4). This analysis involved comparing the changes in Baumann’s angle and the percentage of rotation observed immediately post-operation and at 3 months post-operation, based on different pin configurations (3 lateral pins vs. 2 lateral pins + 1 medial pin). Our findings revealed no significant differences in outcomes between these two pin configurations.

**Figure 4:**
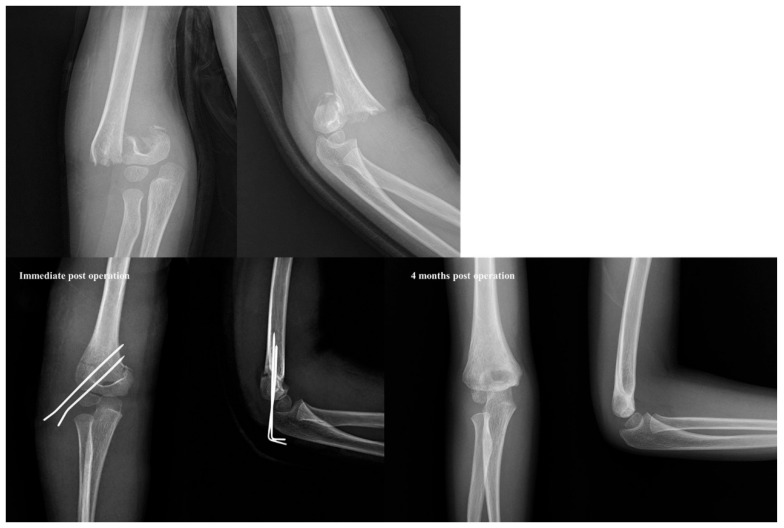
Example of a low transverse fracture type in a 6-year-old boy with a Gartland type III supracondylar fracture. Despite being a Gartland type III fracture, the internal rotational stress test was negative, even with only two lateral pins fixation. Furthermore, there was no loss of reduction in the radiographs taken 4 months post-operation.

**Figure 5:**
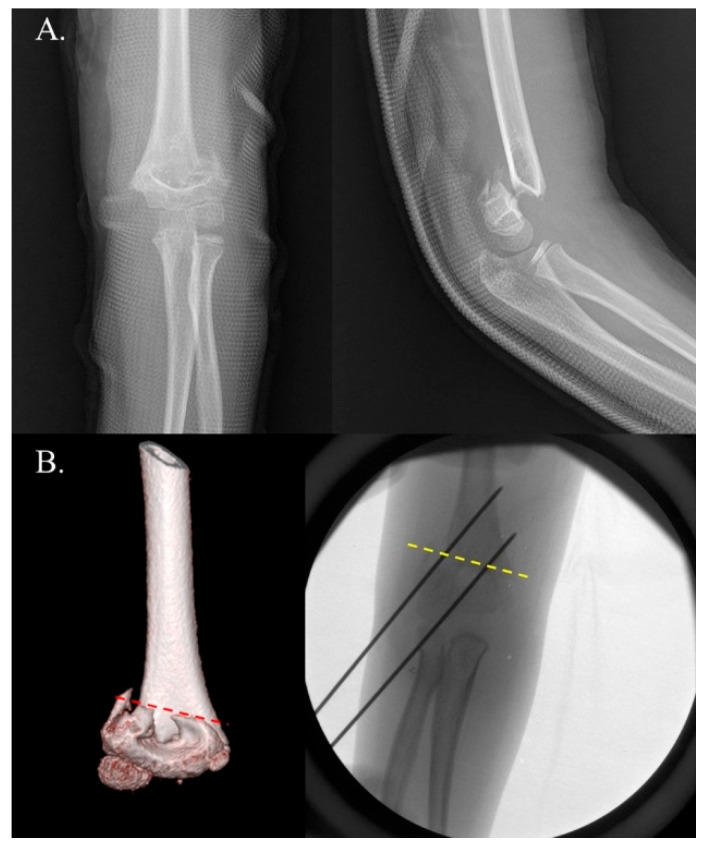
Example of a high oblique fracture on the radial side in a 7-year-old boy with a Gartland type III supracondylar fracture. Panel (**A**) displays the Gartland type III fracture in preoperative radiographs. Panel (**B**) demonstrates that this pattern (red and yellow dashed lines) can be confirmed on preoperative CT scans and C-arm images taken intraoperatively.

**Figure 6:**
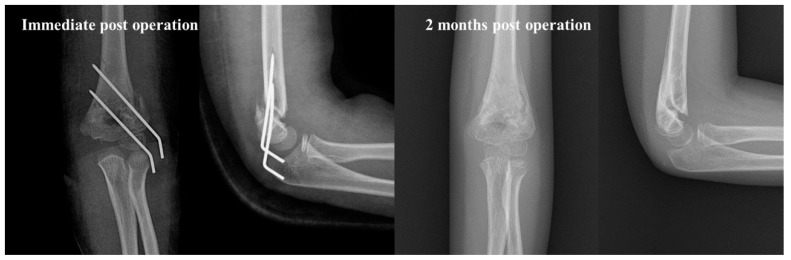
Despite being a Gartland type III fracture, the patient was treated with only two lateral pins and there was no loss of reduction at 2 months post-operation. This configuration increased the angle between the direction of the fracture line and the direction of the pins inserted laterally, resulting in strong fixation.

Our review of the radiographs of the five supracondylar humeral fractures treated with only two lateral pins showed that there were no significant changes in the Bauman angle, HC angle, and rotational percentage immediately after surgery and at 3 months post-operation. This observation led us to identify a fracture pattern that appears to be resistant to rotational forces. In three cases, the fracture line was transverse and located near or below the olecranon fossa (Figure 4). In these instances, the larger contact area of the fracture allowed for strong fixation with only two pins. In the other two cases, the fracture lines were oblique, with the lateral ends being higher (Figure 5). This configuration increased the angle between the direction of the fracture line and the direction of the pins inserted laterally, resulting in strong fixation (Figure 6).

No patient was noted to have a postoperative neurologic deficit that had not been noted before surgery. Two cases of pin-tract infection occurred; Groups 1 and 2 each had one case of superficial infection, respectively. In both instances, it resolved promptly following the removal of the pins.

## 4. Discussion

The distal humerus is particularly vulnerable to instability due to the thin connection between the medial and lateral segments, predisposing it to rotational instability [9,21,22]. This is especially true in cases such as Gartland III or IV fractures, where the periosteum is extensively torn, compromising stability and necessitating more robust fixation to prevent loss of reduction [6,9]. Some suggest that checking for rotational instability during surgery and adding additional pins when necessary is beneficial [5,6,7,9]. The IRST has been reported to reduce the total number of pins used and the incidence of reduction loss when applied during surgery. Nonetheless, there has been no direct comparison between the radiological or clinical outcomes of cases with a positive IRST and those with a negative IRST. Therefore, we initiated this study to compare the radiological and clinical outcomes of two groups based on the IRST results to evaluate the usefulness of IRST.

In our study, no statistically significant differences were observed, either radiologically or clinically, between the two groups, who were IRST positive or negative. Gorden et al. [9] have underscored the importance of ensuring contact, despite the actual bony contact area being small. We achieved adequate anatomical reduction and stability, confirmed by routine external rotation lateral view imaging in all patients. In addition, there were no patients with a major loss of reduction in either group. This demonstrates that the anatomical reduction was not only achieved but also that the fixation was enough to withstand the rotational stress. We believe that if anatomical reduction is achieved and the fixation can handle a certain level of stress (deemed acceptable based on C-arm AP and external rotation lateral views in our study), then the influence of the IRST is considered to be limited in both clinical and radiological outcomes. Zenios et al. [6] stated that the lateral view taken in the operating room is an external view, while the lateral view taken after surgery is an internal view, suggesting that this can give a false impression. This insight prompted the development of the IRST. However, the internal view taken after surgery is not strictly an internal stress test; therefore, it is more accurate to say that the issue is not a failure to withstand rotational stress but incorrect reduction. Moreover, the position required for the test is rarely adopted in daily life after the application of a long arm cast; the significance of IRST results on the postoperative course appears limited if the reduction is adequately achieved during surgery and the fixation can sustain appropriate rotational stress. Given that the IRST is a type of stress test with the potential to cause fixation loss during its execution, we believe that the quality of reduction is more critical than the IRST results themselves. Additionally, we believe it is sufficient if the reduction is deemed acceptable and the strength of the fixation is appropriate, as determined in the operating room using the C-arm for both AP and external rotation views.

Among the patients in Group 1, five individuals were treated with two lateral pins in our cohort. Despite positive results in the IRST for these patients, no significant loss of reduction was observed. Conventionally, Gartland III fractures are thought to possess inherent instability, necessitating stronger fixation [5,6,7,8,9]. This stands in contrast to our findings.

The analysis of the fracture patterns in five patients treated with only two lateral pins led us to hypothesize that certain fracture types are resistant to instability; two types of fracture patterns were observed (Figure 4 and Figure 5): a low transverse pattern and a high oblique pattern on the radial side. The configuration of these fractures suggests that the angle at which the pins are inserted laterally results in a more secure fixation by the lateral pins. Therefore, it is believed that even in cases classified as Gartland type 3, bone union could be achieved without loss of reduction using only two lateral pins, even if the IRST is positive. Conversely, it can be considered that there are fracture patterns vulnerable to instability, and in such types of fractures, it may be beneficial to employ a more robust fixation approach. Further research is necessary to investigate the relationship between the configuration of fractures and the required fixation force.

A total of 31 patients received lateral 3 pin fixation; among these, 19 patients (Group 1) showed positive results in the IRST, while 12 patients (Group 2) were negative. The observation that patients with lateral 3 pins can still test positive in the IRST, and that positive IRST results do not necessarily translate into significant radiological or clinical differences, suggests that appropriate reduction and fixation force can mitigate adverse outcomes. This finding aligns with Skaggs et al. [11], who reported no loss of reduction in their study utilizing only three lateral pins for treatment. The optimal configuration of pins has yet to be established [6,7,9,11]. Biomechanical studies have shown that a cross-configuration offers greater stability than fixation using two parallel lateral pins [23]. In another study, a 2 + 1 configuration was found to be most resistant to rotational forces [24]. Our subgroup analysis did not show a significant difference between the three lateral pins and the two lateral + one medial pin configurations. However, patients with three lateral pins showed positive IRST results (Group 1), whereas those with two lateral pins and one medial pin were all negative in the IRST. This may suggest that the medial pin provides stronger resistance to rotational forces. Nonetheless, the impact of pin configuration on clinical and radiological outcomes requires further investigation.

In our study, Group 2 patients underwent medial pinning based on their IRST results, with eight receiving two lateral pins followed by one medial pin, and two receiving three lateral pins followed by one medial pin. According to Zenios et al. [6], the protocol after inserting two lateral pins involves conducting an IRST, and if positive, adding a lateral third pin, then performing the IRST again; if still positive, a medial pin is then added. However, this approach does not easily account for the configuration of two lateral pins + one medial pin. Moreover, other authors have applied different criteria for the insertion of medial pins [5,7,8]. As our study demonstrates, there are instances where it may not be necessary to insert a third lateral pin after the initial two lateral pins. Therefore, it is deemed necessary to establish clearer criteria for the insertion of medial pins based on the results of the IRST for additional pin fixation.

Our study faced limitations due to its retrospective nature, which inherently carries the risk of selection bias. One potential source of bias is the exclusion of patients without documented IRST results, which may have inadvertently led to a selection of cases where surgeons were more meticulous in documentation. Additionally, differences in individual surgeon preferences for performing IRST or choosing specific pin configurations could have influenced group allocation. While we attempted to standardize patient selection and surgical technique, intraoperative decision-making variations remain a limitation. To minimize bias, IRST was conducted in a consistent and reproducible manner, where the elbow was internally rotated until the surgeon’s palm naturally touched the surgical field. This technique was consistently applied across all cases, ensuring a standardized approach to the test. Additionally, radiographic measurements were performed independently by two orthopedic specialists, and statistical comparisons confirmed that baseline characteristics were similar between groups. Future prospective studies with standardized IRST protocols and randomization are necessary to further validate our findings. Another weakness of our study is the fact that although IRST was performed, patients without record of the test were excluded, potentially introducing selection bias. We acknowledge that Flynn’s score does not comprehensively assess all aspects of functional recovery, such as strength, fine motor control, or patient-reported functional limitations in daily activities. Additionally, the heterogeneity in pin configuration complicates the establishment of a clear control group and comparison. However, our research is significant as it is the first study that compares radiologic and clinical outcomes based on IRST results. Furthermore, we have suggested the possibility that there exist fracture configurations more resistant to instability, even in patients with Gartland type 3 supracondylar fractures.

## 5. Conclusions

In patients with Gartland type III supracondylar fractures, if reduction is adequately achieved and sufficient fixation force is maintained to preserve fixation, which can be determined in the AP and external rotational views on the C-arm, the IRST results do not significantly impact radiologic and clinical outcomes. The degree of fracture reduction and adequate strength of fixation are key to the success of Gartland type III supracondylar fractures. In addition, the pattern of the fracture can influence instability, necessitating further research on this matter. Additionally, at present, there are no validated studies quantifying the forces involved in IRST. Further research regarding whether biomechanical testing could provide further insights into the relevance of IRST is needed.

## Figures and Tables

**Table 1:** Patient pin configurations based on internal rotational stress test results.

	Group 1 (IRST Positive)	Group 2 (IRST Negative)
2 lateral pins	5	0
3 lateral pins	19	12
2 lateral pins +1 medial pin	0	8
3 lateral pins +1 medial pin	0	2
Total	24	22

IRST: internal rotational stress test.

**Table 2:** Comparison of changes in radiographic parameters: Immediate Postop. vs. 3-Month Postop.

	Group 1 (IRST Positive)	Group 2 (IRST Negative)	*p* Value	95% CI
Changes in Baumann’s angle	2.8 ± 2.6	2.6 ± 2.3	0.078 ^†^	(−1.20, 1.55)
Changes in Humerocapitellar angle (deg.)	2.3 ± 2.4	2.2 ± 2.8	0.629 ^†^	(−1.46, 1.68)
Changes in Lateral rotation percentage (%)	3.7 ± 3.0	3.4 ± 3.2	0.769 ^†^	(−1.50, 2.18)

^†^: two-sample *t* test.

**Table 3:** Comparison of Flynn’s score by Group at 1 year post-operation.

	Group 1 (IRST Positive)	Group 2 (IRST Negative)	*p* Value	95% CI
Flynn’s score at 1 year-postoperation			0.8445 ^†^	(−0.90, 1.10)
Excellent	16	14		
Good	5	6		
Fair	3	2		

^†^: Chi-square test.

**Table 4:** Changes in radiographic parameters according to the pin configurations (immediate postop. vs. 3-month postop).

	Patient with 3 Lateral Pins (*n* = 31)	Patient with 2 Lateral Pins + 1 Medial Pin (*n* = 8)	*p* Value	95% CI
Changes in Baumann’s angle	2.90 ± 2.34	2.00 ± 2.39	0.360 ^†^	(−1.35, 1.78)
Changes in Lateral rotation percentage (%)	3.26 ± 2.68	4.63 ± 2.39	0.185 ^†^	(−1.70, 2.45)

^†^: two-sample *t* test.

## Data Availability

The data are not publicly available due to ethical reasons and patient privacy.
